# Misplacement of something inside the refrigerator is not a sign of dementia, but a probable symptom of attention deficit due to depression

**DOI:** 10.1038/s41598-021-84676-x

**Published:** 2021-03-02

**Authors:** Jeewon Suh, So Young Park, Young Ho Park, Jung-Min Pyun, Na-young Ryoo, Min Ju Kang, SangYun Kim

**Affiliations:** 1 Department of Neurology, Seoul National University Bundang Hospital, 82, Gumi-ro 173, Beon-gil, Bundang-gu, Seongnam-si, Gyeonggi-do 13620 Republic of Korea; 2 College of Medicine, Seoul National University, Seoul, Republic of Korea

**Keywords:** Neurology, Signs and symptoms

## Abstract

The objective of this study is to investigate the clinical significance of a specific behavior of misplacing items in a refrigerator (i.e., placing extremely unusual things such as remote control and/or cellular phone in a refrigerator) as a symptom of cognitive dysfunction. Patients with memory complaints were asked whether they ever experienced misplacing items in a refrigerator, such as placing a remote control, a cellular phone, or other extremely unusual things inside a refrigerator (referred to as the ‘fridge sign’). Among the 2172 individuals with memory complaints, 55 (2.5%) experienced symptoms of the ‘fridge sign’. We investigated the cognitive profiles of ‘fridge sign’-positive patients and performed follow-up evaluations with neuropsychological tests or telephone interviews. The ‘fridge sign’ was mostly found in individuals diagnosed as subjective cognitive decline (n = 33, 60%) or mild cognitive impairment (MCI, n = 20, 36.4%) with depressive mood and was relatively rare in dementia states (n = 2, 3.5%). Moreover, none of the ‘fridge sign’-positive patients showed significant cognitive decline over the follow-up period. We compared the cognitive profiles and the clinical progression of 20 ‘fridge sign’-positive MCI patients and 40 ‘fridge sign’-negative MCI patients. ‘Fridge sign’-positive MCI patients had worse scores on the Stroop test color reading and had higher scores on the geriatric depression scale than ‘fridge sign’-negative MCI patients, which indicates that the ‘fridge sign’ could be indicative of selective attention deficit in patients with depression rather than indicative of cognitive decline related to dementia.

## Introduction

As the prevalence of dementia increases in an aging society, there have been growing concerns about dementia. When people visit memory clinics, clinicians interpret the severity of symptoms based on detailed history taking and cognitive function tests. People who visit the clinic with concerns about memory impairment have some degree of experience with misbehavior or mistakes due to memory impairment. These symptoms include placing inappropriate things such as remote controls and/or cellular phones in a refrigerator. Such a scenario has been portrayed in the media—TV drama and movies—as a symbolic behavior of Alzheimer’s disease or dementia. Even many clinicians consider such behavior a ‘red flag sign’ associated with severe cognitive impairment. The fundamental question is whether these behaviors are truly a sign of dementia or of a pre-dementia stage of a neurodegenerative disease. For example, memory decline and attention deficit are often not easy to distinguish and frequently coexist because maintaining appropriate attention is a precondition for storing long-term information^1,2^. Attention deficit may make it difficult to perform memory tasks.

In this study, we investigated the clinical significance of the behavior of misplacing items in a refrigerator (i.e., placing a remote control, a cellular phone, or other extremely unusual things in a refrigerator, henceforth referred to as the ‘fridge sign’) as a symptom of cognitive dysfunction since many people who have had this experience are afraid that they may have dementia.

## Results

Among the 2172 subjects who visited our clinic for the first time with memory complaints, 55 (2.5%) experienced symptoms of the ‘fridge sign’. The demographic findings of ‘fridge sign’-positive patients are shown in Table 1. The ‘fridge sign’-positive patients were composed of 33 with subjective cognitive decline (SCD, 60%), 20 with mild cognitive impairment (MCI, 36.4%) and 2 with dementia due to Alzheimer’s disease (ADD, 3.5%). Among the subjects who never experienced symptoms of the ‘fridge sign’, 557 had SCD, 825 had MCI, 474 had ADD, and the remaining 261 had dementia of other etiologies (vascular dementia, frontotemporal dementia, dementia of Lewy body, etc.). The proportions of SCD and MCI were significantly higher among the ‘fridge sign’-positive patients than among the ‘fridge sign’-negative patients. Forty-three of 55 ‘fridge sign’-positive patients underwent detailed neuropsychological tests. Thirty-three patients (76.74%) had high depression scores according to the 30-item geriatric depression scale (GDepS) score (the cut-off value for a high depression score is > 10). There were no significant differences in demographic profiles between the normal group and the group with high depression scores except age (Table 1). Among the 33 patients with high depression scores, 8 patients were on selective serotonin reuptake inhibitors at initial presentation and 15 patients were not on medication.

**Table 1:** Demographic findings of ‘fridge sign’-positive patients and the comparison between depressive and normal groups according to the Geriatric Depression Scale score.

Characteristics	Total ‘fridge sign’-positive group	Normal group^a^	Depressive group^a^	*P* value*
Number of subjects	55	10	33	
Age	61.07 ± 8.16	66.80 ± 8.70	60.42 ± 7.57	0.021
Female	55 (100%)	10 (100%)	33 (100%)	
Education level, year	10.63 ± 4.84	11.65 ± 5.69	10.03 ± 4.90	0.289
APOE ε4 carrier	5 (9.1%)	1 (10%)	4 (12.1%)	
CDR-SOB	1.22 ± 1.01	0.90 ± 0.32	1.32 ± 0.52	0.430
MMSE	26.35 ± 3.60	26.80 ± 1.69	26.21 ± 3.97	0.764
GDS	2.60 ± 0.62	2.60 ± 0.52	2.70 ± 0.68	0.826
GDepS	17.63 ± 8.93^a^	4.00 ± 2.11	21.76 ± 5.27	< 0.001
**Disease subtype**				0.701
SCD	33 (60%)	5 (50.0%)	17 (51.5%)	
MCI	20 (36.4%)	5 (50.0%)	14 (42.4%)	
Dementia	2 (3.6%)	0 (0.0%)	2 (6.1%)	

Values are given as the mean ± standard deviation or number (%).

*CDR* Clinical Dementia Rating, *CDR-SOB* Clinical Dementia Rating—Sum of box, *MMSE* Mini-Mental Status Examination, *GDS* Global Deterioration Scale, *GDepS* Geriatric Depression Scale, *SCD* subjective cognitive decline, *MCI* mild cognitive impairment.

*P values were obtained using the Mann–Whitney test or Pearson’s chi-squared test between the normal and depressive groups as appropriate.

^a^Only 43 subjects with detailed neuropsychological test scores, including Geriatric Depression Scale scores, were included in the normal and depressive groups.

**Figure 1:**
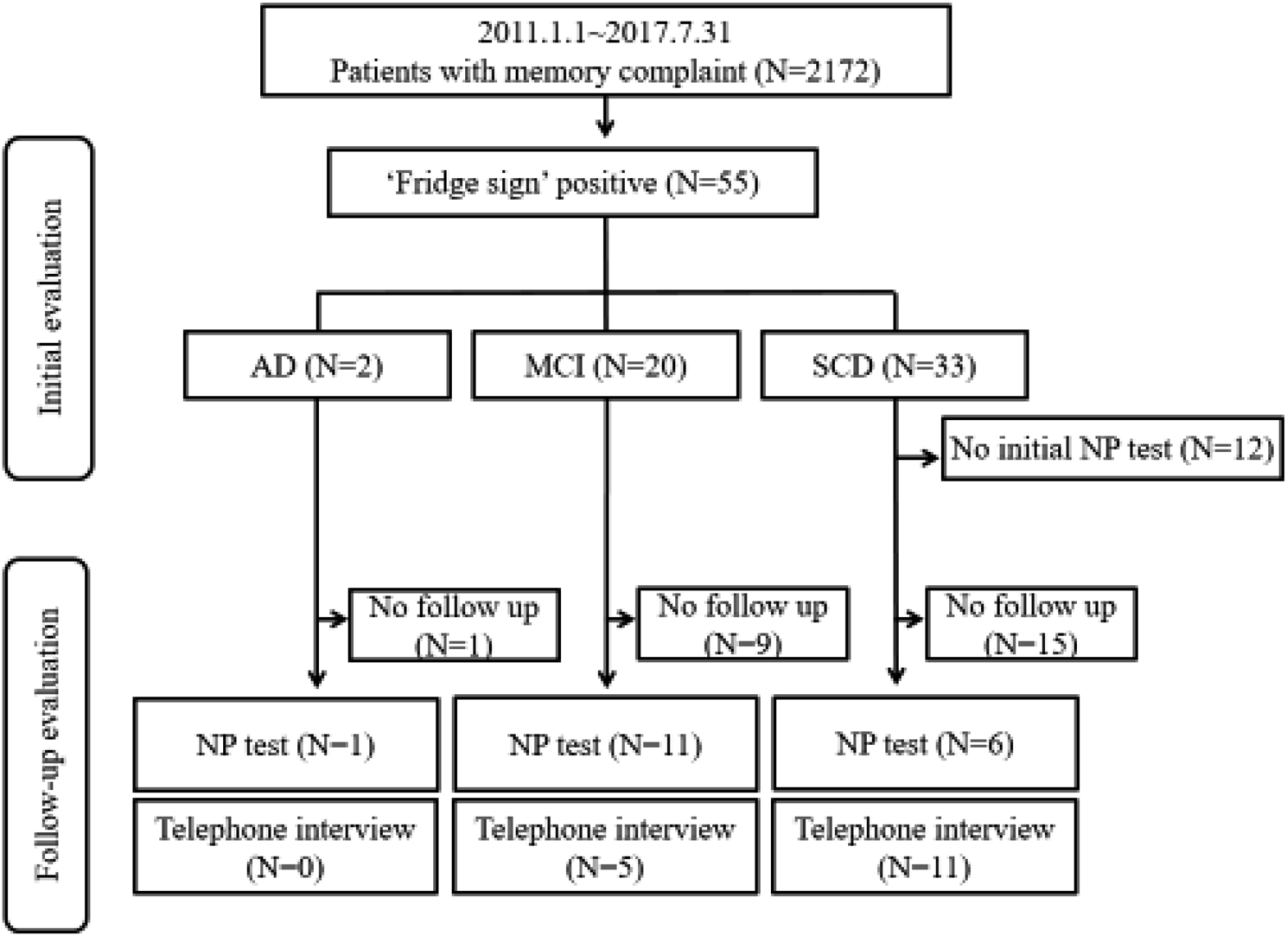
Flow chart of included patients. *SCD* subjective cognitive decline, *MCI* mild cognitive impairment, *AD* Alzheimer’s dementia, *NP test* neuropsychological test. The figure was generated using Microsoft PowerPoint 2013 (https://www.microsoft.com).

**Figure 2:**
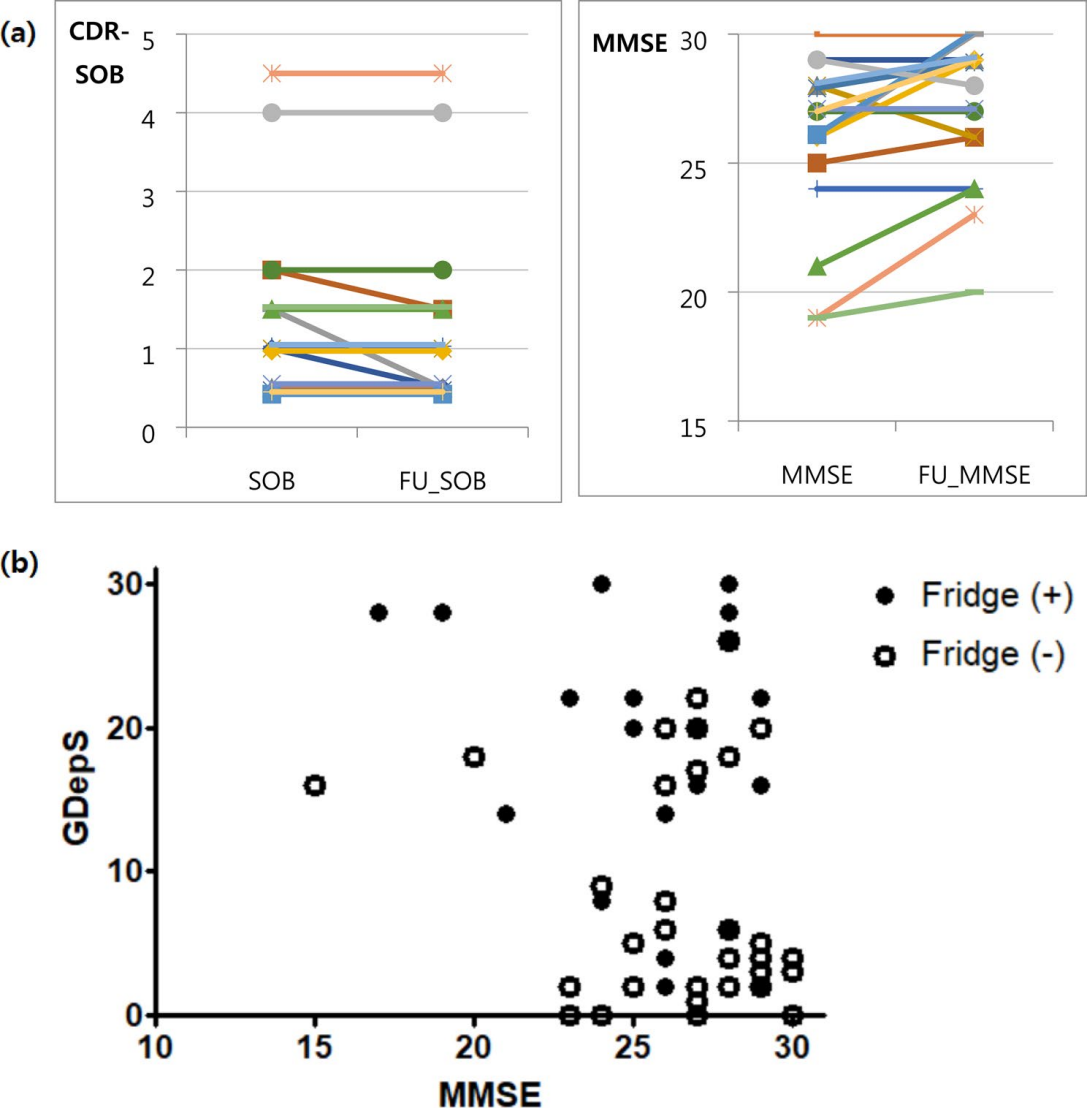
(**a**) Initial and follow-up neuropsychological tests of fifteen patients. (**b**) Distribution of MMSE and Geriatric Depression Scale of MCI patients according to the presence of the fridge sign. *CDR-SOB* Clinical dementia rating-sum of box, *MMSE* Mini-Mental State Examination, *GDepS* Geriatric Depression Scale. The (**a**) was generated using Microsoft Excel 2013 (https://www.microsoft.com) and the (**b**) was generated with GraphPad Prism version 5.01 (https://www.graphpad.com/).

Of the patients with a positive fridge sign, 18 successfully completed the follow-up evaluation regarding their cognitive status, including the neuropsychological test. The mean interval between the initial and follow-up tests was 20 months (range from 8 to 36 months). The patients who were followed-up until the final evaluation were 6 SCD subjects, 11 MCI patients, and one ADD patient (Fig. 1). There was no case showing cognitive decline at the final follow-up evaluation according to the detailed neuropsychological test. Four patients who were initially diagnosed with MCI were ultimately diagnosed with SCD since the final detailed neuropsychological test showed an improvement in cognitive function. The diagnoses of the other 14 patients were not changed. One patient who was diagnosed with ADD with depression showed no disease progression because the Mini-Mental Status Examination (MMSE) score changed from 19 to 20 in 1 year. Eighteen patients showed an improvement in the mean MMSE score, from 25.94 to 27.4. The clinical dementia rating-sum of box (CDR-SOB) improved in three of 18 patients, while it remained the same in 15 patients (Fig. 2a). The mean GDepS score improved from 20.78 points to 14.8 points.

Of the 25 subjects who did not undergo the follow-up neuropsychological test, 16 subjects (64%) responded to the telephone interview. Twelve subjects (9 SCD subjects and 3 MCI patients) showed no change in their cognitive function. Three subjects (one SCD subject and two MCI patients) showed improvement in their cognitive function. Only one subject initially diagnosed with SCD showed cognitive decline after the initial evaluation. The mean Informant Questionnaire on Cognitive Decline in the Elderly (IQCODE) score of all patients was 3.02.

We compared the ‘fridge sign’-positive MCI patients (n = 20) with age-, sex- and education level-matched ‘fridge sign’-negative MCI patients (n = 40) (Table 2). The score of depression (*P* < 0.001) and Stroop test color reading (*P* = 0.018) were significantly different between the two groups. ‘Fridge sign’-positive MCI patients had a worse score on the Stroop test color reading and had a higher GDepS score (i.e., more depressive) than ‘fridge sign’-negative MCI patients. The distribution of the MMSE and GDepS scores between ‘fridge sign’-positive and ‘fridge sign’-negative MCI patients is presented in Fig. 2b. There were no differences in the MMSE, CDR-SOB, or other detailed neuropsychological test results, including digit span, Boston Naming Test (BNT), Rey–Osterrieth complex figure test (RCFT)-copy, phonemic controlled oral word association test (COWAT), verbal learning test (VLT) and trail making tests. Although statistically insignificant, there were tendencies for the ‘fridge sign’-positive MCI patients to have worse scores in digit span forward/backward and VLT recognition scores.

**Table 2:** Comparison of neuropsychological test results between ‘fridge sign’-positive MCI patients and ‘fridge sign’-negative MCI patients.

Variables	Fridge sign (+) MCI (n = 20)	Fridge sign (−) MCI (n = 40)	*P* value*
**Basic demographics**			
Age	60.55 ± 8.89	60.67 ± 7.38	0.954
Education level	10.00 ± 5.15	10.95 ± 3.90	0.429
MMSE	25.45 ± 3.34	26.77 ± 2.95	0.123
CDR-SOB	1.52 ± 0.99	1.34 ± 0.94	0.476
GDepS	17.90 ± 9.46	7.56 ± 7.78	< 0.001
**Neuropsychological test**			
Digit span forward	5.45 ± 1.27	6.12 ± 1.36	0.070
Digit span backward	3.50 ± 1.35	3.98 ± 1.29	0.192
BNT	44.70 ± 9.03	44.63 ± 8.80	0.975
Rey CFT copy	29.30 ± 4.77	30.55 ± 4.33	0.312
VLT delayed recall	5.50 ± 2.28	4.58 ± 3.21	0.255
VLT recognition	19.00 ± 3.55	17.03 ± 5.04	0.123
COWAT	21.00 ± 7.48	23.73 ± 11.17	0.570
Stroop word reading	106.83 ± 15.28	110.92 ± 4.30	0.125
Stroop color reading	71.38 ± 22.88	87.03 ± 22.29	0.018
Trail making test part A	25.21 ± 9.12	21.10 ± 10.51	0.213
Trail making test part B	67.71 ± 70.09	56.58 ± 68.83	0.620

Values are given as the mean ± standard deviation.

*MCI* mild cognitive impairment, *MMSE* Mini-Mental State Examination, *CDR* clinical dementia rating, *SOB* sum of box, *GDS* geriatric depression scale, *BNT* Boston Naming Test, *Rey CFT* Rey Complex Figure Test, *SVLT* verbal learning test, *COWAT* Controlled Oral Word Association Test.

*P values were obtained using the Mann–Whitney test.

Among the included MCI patients, 16 ‘fridge sign’-positive patients and 40 ‘fridge sign’-negative patients were undergoing follow-up evaluation either by detailed neuropsychological tests or structured telephone interviews (Table 3). Seven patients among the ‘fridge sign’-negative patients progressed to dementia; in contrast, none of the ‘fridge sign’-positive patients showed progression of cognitive decline.

**Table 3:** Comparison of the change in cognitive function between ‘fridge sign’-positive MCI patients and ‘fridge sign’-negative MCI patients.

Fridge sign (+) MCI		Fridge sign (−) MCI	
f/u diagnosis	N (%)	f/u diagnosis	N (%)
**Detailed neuropsychological test**			
Normal	4 (36.4%)	Normal	1 (2.7%)
MCI	7 (63.6%)	MCI	29 (78.4%)
Dementia	0 (0.0%)	Dementia	7 (18.9%)
**Telephone interview**			
Improved	2 (40%)	Improved	0 (0.0%)
No change	3 (60%)	No change	3 (100%)
Worsened	0 (0.0%)	Worsened	0 (0.0%)

Telephone interviews were evaluated by the IQCODE with a cut-off value < 3.00 indicative of improvement and > 3.44 indicative of worse cognitive function.

*MCI* mild cognitive impairment.

## Discussion

People often think of the ‘fridge sign’ as a symptom of dementia and memory impairment, and even some physicians consider the symptom a representative red flag for dementia. Contrary to common stereotypes, the ‘fridge sign’ is a relatively rare condition among individuals with memory complaints. In particular, the ‘fridge sign’ is mostly found in individuals diagnosed with SCD or MCI and is relatively rare in dementia states.

A conspicuous aspect of patients who visited our memory clinic with the ‘fridge sign’ was that they were more depressive and inattentive subjects with or without cognitive impairment. According to the detailed neuropsychological test, there was a significant difference in the Stroop color reading test score between the ‘fridge sign’-positive and ‘fridge sign’-negative patients. Depression scores were also significantly different between the two groups. Therefore, we can assume that the ‘fridge sign’ is one of the signs indicating attention deficit due to depressive symptoms, whilst its presence does not exclude the presence of a pre-dementia or dementia syndrome.

Over the past decade, many studies have demonstrated a decrease in cognitive function in depressed patients^3–5^. To date, the affected cognitive domains in patients with depression have been extensively identified as psychomotor speed^6^, short-term and explicit long-term memory^7,8^, and executive function, including attention^9,10^. The change in attention in depression is worth noting because it is consistently confirmed to be reduced in depressed patients, acting as a gatekeeper of general cognitive function^1^.

The Stroop test measures selective attention^11–13^ and depressed patients perform poorly on Stroop tests^3,14^. Overall, we can assume that patients with the ‘fridge sign’ may have decreased selective attention due to depression. There was no statistically significant difference, but patients with the ‘fridge sign’ showed poor performance on the digit span forward and backward tests. These results also indicate that the characteristic cognitive profile of patients with the ‘fridge sign’ is a decrease in selective attention. The symptom of putting inappropriate items inside the refrigerator may be interpreted as a goal neglect response from interference in conflict situations, which is a symbolic expression of attention deficit associated with depressive mood, not with dementia or other specific cognitive dysfunction, as it has been commonly misunderstood by the general public and some clinicians. The ‘fridge sign’ in depressive patients with attention deficit seems to be explained as an automatic or habitual action, instead of purposeful work.

According to our observations throughout the follow-up period of 2 years, patients with the ‘fridge sign’ did not show progressive cognitive decline, according to the neuropsychological test or telephone interview; rather, they showed an improvement in both cognitive and functional status, especially in patients with reduced depression scores after anti-depressant treatment. In a comparison of ‘fridge sign’-positive and ‘fridge sign’-negative MCI patients, 18.9% of the ‘fridge sign’-negative patients progressed to dementia, while none of the ‘fridge sign’-positive patients progressed to dementia. Therefore, it can be said that the ‘fridge sign’ has little relation to the progressive degenerative cognitive impairment.

This study has potential limitations. First, this investigation was a single-center study, and a very small number of people with memory complaints presented the ‘fridge sign’. Due to the low occurrence of the ‘fridge sign’, only a small number of subjects were included in subsequent analysis. In addition, the ‘fridge sign’ could only be measured by asking the subjects or their caregivers about their experience. So, there was a possibility of overrepresentation of SCD subjects reporting the ‘fridge sign’, whilst the patients with dementia could not report such symptoms due to their memory deficits, even though the investigation included the reports from caregivers or families. Therefore, the results of this study are preliminary and need further investigation to validate the clinical significance of the ‘fridge sign’. Second, more than half of the patients with the ‘fridge sign’ did not undergo the follow-up neuropsychological test. We conducted telephone interviews with a structured questionnaire to address this shortcoming. However, detailed neuropsychological tests are essential to accurately understand the cognitive changes in patients with the ‘fridge sign’. Third, we defined and analyzed only the specific symptoms of putting inappropriate items in the refrigerator, referring to the phenomenon of putting things in a very inappropriate place. Since women are more familiar with the refrigerator and other kitchen equipment than men are, only female patients showed the ‘fridge sign’ in our study. For a better understanding of the phenomenon of the ‘fridge sign’ in both men and women, a different definition of the ‘fridge sign’ will be needed.

The fridge sign is a rare behavior among people who complain of cognitive decline, though it has a large impact on patients, caregivers, and sometimes physicians. Through our study, we found that the ‘fridge sign’ is not a key feature of degenerative cognitive disease but rather a goal neglect response due to the selective attention deficit influenced by depressive mood. If a person complains of the ‘fridge sign’ with cognitive decline, evaluating the patient’s attention function and measuring the degree of depression would be important steps for the accurate diagnosis and treatment of cognitive dysfunction.

## Methods

### Participants

We collected data from subjects who visited the Neurocognitive Behavior Center of Seoul National University Bundang Hospital with memory complaints between January 2011 and July 2017. At the initial visit, we asked all subjects and their caregivers whether they ever experienced misplacing items in a refrigerator, such as placing a remote control, a cellular phone, or other extremely unusual things in a refrigerator. Detailed neuropsychological tests were performed to identify the cognitive profiles. The diagnoses of the included subjects were determined by expert neurologists. The diagnosis of SCD was based on the criteria proposed by Jessen et al.^15^, and the diagnosis of MCI was based on the criteria proposed by Peterson et al.^16^. ADD was diagnosed by the criteria proposed by the National Institute on Aging and Alzheimer’s Association^17^. Moreover, we compared patients with ‘fridge sign’-positive MCI and those with ‘fridge sign’-negative MCI. ‘Fridge sign’-negative MCI patients were defined as those who were matched in age, sex, and education level with the ‘fridge sign’-positive MCI patients who visited our clinic with memory complaints during the same period and were diagnosed with MCI but without experience of the ‘fridge sign’. We excluded the data of people on medication with the anticholinergic effects and on benzodiazepines. The protocol for the study was approved by the Institutional Review Board (IRB) of the Seoul National University Bundang Hospital and conformed to the provisions of the Declaration of Helsinki. All subjects’ anonymity was preserved. Informed consent was waived for this retrospective study which was approved by the IRB of the Seoul National University Bundang Hospital.

### Neuropsychological assessment

We evaluated attention, language function, visuospatial function, verbal and visual memory, and frontal/executive function using a series of standardized detailed neuropsychological tests. We collected the results of digit span test(forward and backward)^18^, Boston Naming Test (BNT)^19^, Rey–Osterrieth complex figure test (RCFT, copy)^20^, verbal learning test (VLT)^21^ composed of free recall trials of 12 words, 20-min delayed recall task, recognition task, phonemic controlled oral word association test (COWAT)^22^, Stroop test^23^ (word and color reading of 112 items within 2 min), and Trail making test part A and B^24^. The depression score was measured according to the self-reported 30-item geriatric depression scale (GDepS)^25^.

### Follow-up evaluation

We collected follow-up neuropsychological data from the included subjects. For individuals who had not undergone the follow-up neuropsychological test, we conducted telephone interviews to assess changes in cognitive function. Cognitive changes were evaluated using the Informant Questionnaire on Cognitive Decline in the Elderly (IQCODE)^26,27^. We used the cut-off value of ≥ 3.44 as the cognitive decline and below 3.00 as improvement of cognitive function after the initial IQCODE evaluation^28^.

### Statistical analyses

The data were analyzed using SPSS 20.0. The Mann–Whitney U test was used to compare age, education level, mini-mental state examination (MMSE)^29^, clinical dementia rating (CDR)^30^, CDR—sum of box (CDR-SOB), GDepS, and all scores of neuropsychological tests between the ‘fridge sign’-positive and ‘fridge sign’-negative MCI groups.

## Data Availability

The data supporting the findings of this study are available from the corresponding author upon request.
